# Single-Nuclei RNA Sequencing Shows the Engagement of PPAR-Delta Target Genes Primarily in Hepatocytes and Cholangiocytes by the Selective PPAR-Delta Agonist Seladelpar

**DOI:** 10.1155/ppar/2935230

**Published:** 2025-10-23

**Authors:** Tomoo Yamazaki, Yongqiang Yang, David Schöler, Yoshimi Yukawa-Muto, Tetsuya Kouno, Aenne Harberts, Sadatsugu Sakane, Linton Freund, Cynthia L. Hsu, Thomas C. Whisenant, Sara Brin Rosenthal, Tatiana Kisseleva, Edward E. Cable, Bernd Schnabl

**Affiliations:** ^1^Department of Medicine, University of California San Diego, La Jolla, California, USA; ^2^Department of Medicine, Division of Gastroenterology and Hepatology, Shinshu University, Matsumoto, Japan; ^3^Department of Surgery, University of California San Diego, San Diego, California, USA; ^4^Department of Surgery, University of California San Diego, La Jolla, California, USA; ^5^CymaBay Therapeutics, Fremont, California, USA; ^6^Department of Medicine, VA San Diego Healthcare System, San Diego, California, USA

**Keywords:** liver nonparenchymal cells, metabolic dysfunction–associated steatohepatitis, peroxisome proliferator–activated receptors, primary biliary cholangitis, RNA sequencing

## Abstract

**Background and Aims:**

The selective peroxisome proliferator–activated receptor delta (PPARD) agonist seladelpar reduces liver injury and modulates bile acid metabolism in preclinical models. Seladelpar was recently approved for the secondary treatment of primary biliary cholangitis (PBC). Despite its beneficial effects for liver diseases, the target cells of seladelpar on a single-cell level remain unknown. This study is aimed at investigating the effect of seladelpar on single liver cells.

**Methods and Results:**

CD-1 mice were gavaged with vehicle or seladelpar (10 mg/kg body weight), and the liver was harvested 6 h later. Single-nuclei RNA sequencing (snRNA-seq) analysis showed the engagement of PPARD target genes primarily in hepatocytes and cholangiocytes by seladelpar. The top two upregulated genes, *Ehhadh* and *Cyp4a14*, are related to fatty acid metabolism and were increased in hepatocytes, cholangiocytes, and Kupffer cells. *Abcb4*, an important canalicular transporter with hepatoprotective effects, was significantly upregulated in hepatocytes. We confirmed upregulated *Abcb4* gene expression in seladelpar-treated primary mouse hepatocytes isolated from C57BL/6 mice. We further incubated nonparenchymal liver cells with seladelpar. Although there was a significant increase in the PPARD-responsive genes *Pdk4* and *Angptl4* in cholangiocytes, Kupffer cells, and hepatic stellate cells, seladelpar did not exert specific liver-protective effects in these cell types.

**Conclusion:**

The selective PPARD agonist seladelpar induced PPARD-responsive genes primarily in hepatocytes and cholangiocytes. Seladelpar upregulated *Abcb4* in hepatocytes, which might contribute to its beneficial effects in cholestatic liver disorders.

## 1. Introduction

Peroxisome proliferator–activated receptors (PPARs) are nuclear receptors involved in transcriptional regulation and play an important role in many physiological and metabolic processes [1]. The binding of various endogenous or exogenous lipophilic ligands to PPARs facilitates interaction with coactivator complexes and initiation of transcription [2, 3]. In the liver, the central organ of metabolism, PPARs are involved not only in the regulation of energy and glucose metabolism, but they also have anti-inflammatory properties, inhibit bile acid secretion, regulate endoplasmic reticulum stress, and inhibit carcinogenesis [4].

Three distinct subtypes of PPARs, PPAR-alpha (PPARA), PPAR-delta (PPARD), and PPAR-gamma (PPARG), are known, but the detailed function of PPARD in the liver, which is ubiquitously expressed in all organs, is not well understood [5, 6]. In our prior work, we found that the selective PPARD agonist seladelpar ameliorates ethanol-induced liver disease by modulating bile acid metabolism in a preclinical model [7]. Seladelpar suppressed bile acid synthesis by decreasing Cholesterol 7 alpha-hydroxylase (CYP7A1) through the Fibroblast Growth Factor 21 (FGF21) signaling pathway in mouse and human hepatocytes [8]. From a clinical perspective, seladelpar has shown promising clinical efficacy as a secondary treatment for primary biliary cholangitis (PBC), a chronic cholestatic liver disease, and was recently conditionally approved by the US Food and Drug Administration (FDA) [9, 10]. Despite its clear clinical benefit, the direct effects of seladelpar on individual liver cells remain unclear. This study is aimed at clarifying the effect of seladelpar on a single-cell level in the liver of mice and confirming the induction of PPARD-responsive genes by isolating different types of liver cells.

## 2. Methods

### 2.1. Reagents

Seladelpar (MBX-8025) was provided by CymaBay Therapeutics. Lipopolysaccharide (LPS) was purchased from Enzo Life Sciences (ALX-581-011-L002). PE antimouse CD326 (EpCAM) antibody was obtained from BioLegend (118206). Recombinant Human TGF-*β*1 was purchased from Protein Tech (HZ-1131).

### 2.2. Mice

For single-nuclei RNA sequencing (snRNA-seq), wild-type male CD-1 mice were gavaged with vehicle (PBS) or seladelpar (10 mg/kg body weight), and the liver was harvested 6 h later. Mice had free access to food and water and were maintained on a 12-h artificial light and dark cycle. CD-1 mouse studies were performed at Bayside and approved by Bayside BioSciences IACUC (Santa Clara, CA), approval number BaysideBio 2023-002. CD-1 mice and the 6-h time point were selected since previous data in C57BL/6 mice demonstrated early responses at 1 h, and the objective was to assess direct target engagement and not subsequent changes in physiology [11]. The animal protocol adhered to the 2020 AVMA Guidelines for the Euthanasia of Animals, specifically utilizing exsanguination and organ collection under 4%–5% isoflurane anesthesia.

For bulk RNA sequencing experiments, wild-type C57BL/6 mice were purchased from Charles River and housed in a vivarium of the University of California San Diego (UCSD). C57BL/6 mouse studies were performed at UCSD. Male mice were gavaged with vehicle (PBS) or seladelpar (10 mg/kg body weight), and the liver was harvested 6 h later. All animal procedures, including euthanasia, were conducted in accordance with the guidelines approved by the Institutional Animal Care and Use Committee (IACUC) of the UCSD (protocol S09042). Euthanasia was performed either utilizing exsanguination and organ collection under anesthesia or using gradual fill of carbon dioxide (CO_2_) into the home cage at a displacement rate of 10%–30% of the chamber volume per minute, in accordance with the American Veterinary Medical Association (AVMA) Guidelines for the Euthanasia of Animals and institutional protocols to minimize animal distress.

### 2.3. snRNA-Seq and Bulk RNA Sequencing

Sections of liver (~100 mg) were removed from the mice and snap frozen in liquid nitrogen at Bayside Bio. Frozen livers were delivered to SeqMatic (Fremont, California), where all the subsequent procedures were performed. Nuclei from a portion (~10 mg) of the sample were isolated using the NP-40 lysis method [12]. The number of nuclei was counted using the Countess II QC procedure, and the volume containing 100,000 nuclei was ascertained. Nuclei were fixed using the Parse fixation protocol (https://support.parsebiosciences.com/hc/en-us/article_attachments/24507664016916). The library was prepped from 100,000 parse-fixed nuclei (https://support.parsebiosciences.com/hc/en-us/article_attachments/26024923251348) and sequenced on an Illumina NovaSeq X Plus 300-cycle sequencer. Fastq files were run through the Parse Biosciences tool parse-splitpipe, which runs an alignment algorithm (aligned to the mouse genome mm10) and outputs cell × gene count matrices. SoupX (V1.6.2) [13] was run on the output files from the Parse Biosciences split-pipe pipeline, in order to reduce the effect of contaminating ambient RNA (ambient RNA from hepatocytes that split during prep is common in liver single-cell applications). Following SoupX, standard Seurat preprocessing steps were performed, including filtering low-quality cells (cells were removed if they had more than 5% mitochondrial counts, fewer than 200 features, or more than 6000 features). Further cells were removed if they were predicted to be doublets (after running DoubletFinder) [14]. Following preprocessing, the data were normalized and integrated with Harmony, UMAP dimensionality reduction was performed, and clusters were identified using Seurat. Clusters were annotated by examining the expression of known liver cell marker genes within each cluster (using PanglaoDB as a reference) [15]. Differential analysis between seladelpar and vehicle was conducted in each liver cell type separately, using the pseudobulk functionality provided by Seurat (using the AggregateExpression function). Counts from each sample in each cluster were aggregated together, and the differential gene expression analysis was conducted on these aggregated counts, using DeSeq2, implemented as a method in Seurat's FindMarkers function. There were five individual mice per condition. It has been shown that using such a pseudobulk approach helps to minimize false positives in single-cell differential expression analysis [16, 17].

For bulk RNA sequencing, livers from vehicle (*n* = 6) and seladelpar-treated mice (*n* = 7) were used. Primary hepatocytes treated with vehicle (*n* = 9) or seladelpar (10 *μ*M) (*n* = 9) for 48 h were also used. Total RNA was extracted using TRIzol (Invitrogen). Gene expression values were generated following alignment of reads to the mouse genome (GRCm39) using the STAR V2.5.1a aligner [18] and estimation of gene-level counts using RSEM V1.3.0 [19]. BioConductor packages edgeR [20] and Limma [21] implemented within the R statistical computing environment (http://www.cran.org/) were used to implement trimmed mean of *M*-values (TMM) normalization [22] and the limma–voom method [21] for differential expression analysis. Significance was defined by an adjusted *p* value < 0.05 after multiple testing corrections using a moderated *t*-statistic in Limma.

### 2.4. Isolation and Culture of Primary Mouse Hepatocytes

Primary mouse hepatocytes were isolated from 9- to 15-week-old C57BL/6 mice. After mice were anesthetized using a ketamine/xylazine mixture, the vena cava was cannulated, and the liver was perfused for 5 min at 10 mL/min with SC-1 buffer, followed by the SC-2 buffer containing Collagenase D and Collagenase P (Roche Diagnostics) at 10 mL/min for 7 min. The liver was dissected from mice, and the capsule was ruptured with a forceps in Dulbecco's Modified Eagle's Medium (DMEM) containing 10% fetal bovine serum (FBS). The cells were filtered through a 70-*μ*m cell strainer. Hepatocytes were obtained by centrifugation at 84 g for 1 min. Isolated cells were plated in 6-well collagen-coated plates in DMEM containing 10% FBS, 0.35 *μ*M insulin, and 0.1 *μ*M dexamethasone. After 3-h attachment, the cell medium was changed, and cells were treated with seladelpar (10 *μ*M) in the medium with 10% FBS. RNA samples were collected after 48 h of treatment with seladelpar. Isolation and culture were conducted as previously described [8].

### 2.5. Isolation and Culture of Primary Mouse Cholangiocytes

Primary mouse cholangiocytes were isolated from 12- to 15-week-old C57BL/6 male mice. After mice were anesthetized using a ketamine/xylazine mixture, the vena cava was cannulated. The liver was perfused for 6 min at 5 mL/min with DMEM F12 containing Collagenase Type IV (Sigma-Aldrich) and 1% FBS. The liver was excised and incubated in DMEM F12 containing Collagenase Type IV, hyaluronidase (Sigma-Aldrich), and DNase I (Roche) at 37°C for 30 min. Then, 33% FBS was added to the samples to deactivate the enzyme reaction and then filtered through a 100-*μ*m cell strainer. After washing and centrifugation, cholangiocytes were isolated from the second layer of a three-layer discontinuous density centrifugation gradient with 26.25%, 41.5%, and 73.3% Percoll (Cytiva). In addition, EpCAM-positive cells were purified using the PE antimouse EpCAM antibody (BioLegend 118206), EasySep Release Mouse PE Positive Selection Kit (Stem Cell Technology 17656), and EasyPlate EasySep Magnet (Stem Cell Technology 18102). The purity of the cell samples after enrichment was confirmed by fluorescence-activated cell sorting (FACS) and quantitative PCR (Figure S1). Gene expression of Keratin 19 (*Krt19*) and Keratin 7 (*Krt7*) was significantly higher in cholangiocytes compared with hepatocytes. Cholangiocyte samples were cultured with DMEM F12 medium containing 10% FBS, 1% antibiotic–antimycotic (Gibco), 50 mg/mL gentamicin, 1 *μ*M dexamethasone, 3.4 mg/mL triiodothyronine, 10 *μ*g/mL mEGF, and 0.411 mg/mL forskolin and used for experiments after three to four passages. The cell isolation and culture processes were conducted as previously reported [23]. Cholangiocytes were incubated with seladelpar (10 *μ*M) for 8, 24, and 48 h. LPS (100 or 200 ng/mL) was added in the presence or absence of seladelpar for 6 days.

### 2.6. Isolation and Culture of Primary Mouse Kupffer Cells (KCs) and Hepatic Stellate Cells (HSCs)

Primary mouse KCs and HSCs were isolated from 15- to 20-week-old C57BL/6 male mice. After mice were anesthetized using a ketamine/xylazine mixture, the vena cava was cannulated. The liver was perfused for 5 min at 10 mL/min with SC-1 buffer, followed by the SC-2 buffer containing pronase (Roche) at 10 mL/min for 5 min and the SC-2 buffer containing Collagenase Type IV (Sigma-Aldrich) at 10 mL/min for 5 min. Digested livers were excised from mice and incubated for 15 min in SC-2 buffer containing pronase and collagenase, as well as DNase I (Roche). Cells were filtered through a 70-*μ*m cell strainer and then centrifuged. KCs and HSCs were isolated from the three-layer discontinuous density centrifugation gradient with 8.2% (wt/vol) and 14.5% (wt/vol) Nycodenz (Axis-Shield). HSCs were collected from the first layer, and KCs were collected from the second layer. After harvesting, each cell was seeded into plates; concentrations of 1.0–1.5 × 10^6^ cells/mL were used for KCs. Roswell Park Memorial Institute (RPMI) medium containing 10% FBS and antibiotics was used for KCs, and DMEM containing 10% FBS and antibiotics was used for HSCs. Isolation and culture were conducted as previously described [24]. Seeded cells were incubated overnight before treatment with seladelpar (10 *μ*M). KCs were incubated with seladelpar for 2 h, before LPS (100 ng/mL) was added for an additional 6 h. HSCs were incubated on Culture Day 2 with seladelpar (10 *μ*M) for 48 h and then harvested.

### 2.7. Isolation and Culture of Primary Human HSCs

Liver cells were isolated as described [25, 26]. Human HSCs were seeded in 6-well plates and cultured for 2 days under standard conditions. Cells were pretreated with 10 *μ*M seladelpar for 2 h prior to stimulation. Human TGF-*β*1 stimulation (5 ng/mL) was performed for 24 h. Deidentified donor livers (IRB 171883XX, certified by HRPP Director and IRB Chair at UCSD as “no human subjects” according to the Code of Federal Regulations, Title 45, Part 46, and UCSD Standard Operating Policies and Procedures) were obtained via Lifesharing OPO.

### 2.8. Real-Time Quantitative PCR

Total RNA was extracted from frozen tissue or primary cells using TRIzol (Invitrogen). Complementary DNAs (cDNAs) were generated using the high-capacity cDNA reverse transcription kit (Applied Biosystems). The cDNA was amplified and quantified using SYBR Green (Bio-Rad Laboratories). Relative gene expression was determined by CT value and normalized to mouse 18s or human TBP as the housekeeping gene. Primer sequences are listed below:

Mouse *18*s forward AGTCCCTGCCCTTTGTACACA

Mouse *18s* reverse CGATCCGAGGGCCTCACTA

Mouse ATP-binding cassette, Subfamily B, Member 4 (*Abcb4*) forward ACGGGCTCTTGACTTACCAC

Mouse *Abcb4* reverse CTACGACCCCACAGAGGGTA

Mouse cytochrome P450, Family 4, Subfamily a, Polypeptide 14 (*Cyp4a14*) forward TTTAGCCCTACAAGGTACTTGGA

Mouse *Cyp4a14* reverse GCAGCCACTGCCTTCGTAA

Mouse enoyl-CoA hydratase and 3-hydroxyacyl CoA dehydrogenase (*Ehhadh*) forward ATGGCTGAGTATCTGAGGCTG

Mouse *Ehhadh* reverse GGTCCAAACTAGCTTTCTGGAG

Mouse Pyruvate Dehydrogenase Kinase 4 (*Pdk4*) forward GGGTCTCAATAGTGTCACC

Mouse *Pdk4* reverse GTGGGCCTGGGCATTTAGCA

Mouse Angiopoietin-Like 4 (*Angptl4*) forward AAGATGACCCAGCTCATTGG

Mouse *Angptl4* reverse GGAAAAGTCCACTGTGCCTC

Mouse Solute Carrier Family 4, Member 2 (*Slc4a2*) forward GGCGCAGATTCTTTGCACAC

Mouse *Slc4a2* reverse TCCACACCTAAGGTACGAAGTT

Mouse Interleukin 6 (*Il6*) forward TGATGCACTTGCAGAAAACA

Mouse *Il6* reverse ACCAGAGGAAATTTTCAATAG

Mouse C–C Motif Chemokine Ligand 2 (*Ccl2*) forward ATTGGGATCATCTTGCTGGT

Mouse *Ccl2* reverse CCTGCTGTTCACAGTTGCC

Mouse tumor necrosis factor alpha (*Tnfa*) forward AGGGTCTGGGCCATAGAACT

Mouse *Tnfa* reverse CCACCACGCTCTTCTGTCTAC

Mouse Interleukin-1 beta (*Il1b*) forward GGTCAAAGGTTTGGAAGCAG

Mouse *Il1b* reverse TGTGAAATGCCACCTTTTGA

Mouse C-X-C Motif Chemokine Ligand 1 (*Cxcl1*) forward TGCACCCAAACCGAAGTC

Mouse *Cxcl1* reverse GTCAGAAGCCAGCGTTCACC

Mouse C-X-C Motif Chemokine Ligand 2 (*Cxcl2*) forward AAAGTTTGCCTTGACCCTGAA

Mouse *Cxcl2* reverse CTCAGACAGCGAGGCACATC

Mouse Transforming Growth Factor Beta 1 (*Tgfb1*) forward GGAGAGCCCTGGATACCAAC

Mouse *Tgfb1* reverse CAACCCAGGTCCTTCCTAAA

Mouse Collagen Type I Alpha 1 (*Col1a1*) forward TAGGCCATTGTGTATGCAGC

Mouse *Col1a1* reverse ACATGTTCAGCTTTGTGGACC

Mouse actin alpha cardiac/smooth muscle (*Acta2*) forward GTCCCAGACATCAGGGAGTAA

Mouse *Acta2* reverse TCTATCGGATACTTCAGCGTCA

Mouse tissue inhibitor of Metalloproteinase 1 (*Timp1*) forward AGGTGGTCTCGTTGATTTCT

Mouse *Timp1* reverse GTAAGGCCTGTAGCTGTGCC

Mouse *Ccl5* forward GCTGCTTTGCCTACCTCTCC

Mouse *Ccl5* reverse TCGAGTGACAAACACGACTGC

Mouse *Krt19* forward AGGTCAGTGTGGAGGTGGATTC

Mouse *Krt19* reverse GTTCAGCTCCTCAATCCGAG

Mouse *Krt7* forward AGGAGATCAACCGACGCAC

Mouse *Krt7* reverse GTCTCGTGAAGGGTCTTGAGG

Human *TBP* forward GAGCTGTGATGTGAAGTTTCC

Human *TBP* reverse TCTGGGTTTGATCATTCTGTAG

Human *COL1A1* forward AAGAGGAAGGCCAAGTCGAG

Human *COL1A1* reverse CACACGTCTCGGTCATGGTA

Human *ACTA2* forward AAAAGACAGCTACGTGGGTGA

Human *ACTA2* reverse GCCATGTTCTATCGGGTACTTC

### 2.9. Statistical Analysis

All data are expressed as mean ± S.E.M. For comparison of two groups, an unpaired Student's *t*-test was performed. For comparison of multiple groups within one experimental setting, one-way ANOVA with Dunnett's post hoc test or Tukey's post hoc test was conducted. Statistical analyses were performed with GraphPad Prism (V10.4.1). A *p* value < 0.05 was considered significant.

## 3. Results

### 3.1. snRNA-Seq Shows Engagement of Seladelpar Mainly in Hepatocytes and Cholangiocytes

**Figure 1:**
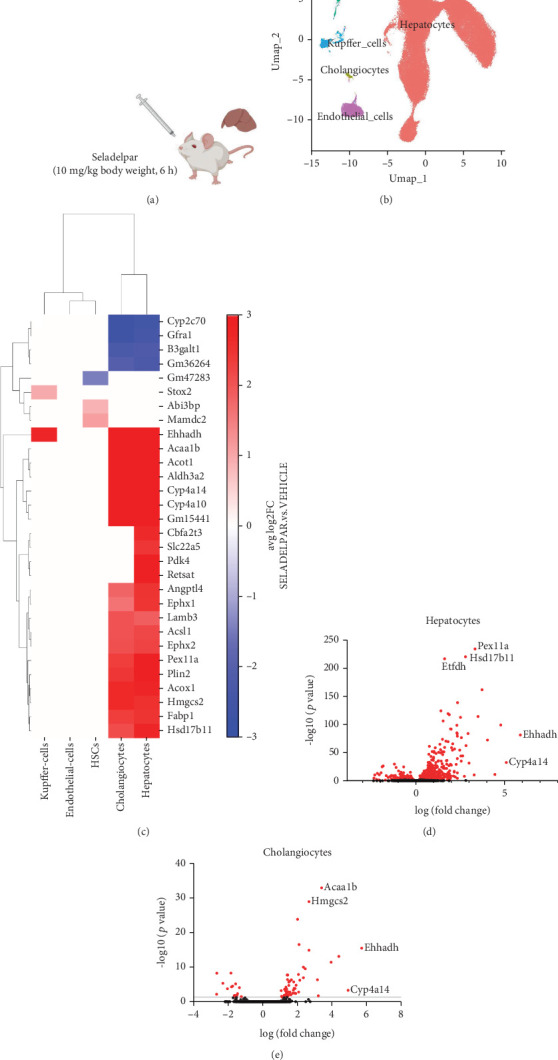
snRNA sequencing and gene induction by seladelpar in liver cells. (a) Male wild-type CD-1 mice (*n* = 5) were gavaged with seladelpar (10 mg/kg body weight) and harvested after 6 h. (b) UMAP visualization of single cells profiled in this study. Each dot represents a cell that is color-coded by cell type. (c) Heat map representing the top 20 genes induced by seladelpar in each liver cell. The number of significantly differentially regulated genes by treatment with seladelpar was 733 (upregulated: 560, downregulated: 173) in hepatocytes, 6 in cholangiocytes (up: 51, down: 15), 2 in KCs (up: 2, down: 0), 3 in HSCs (up: 2, down: 1), and 0 in LSECs. (d) Volcano plot of differential gene expression analysis on hepatocytes between vehicle and seladelpar. Significantly upregulated and downregulated genes in the seladelpar group are shown in red dots. (e) Volcano plot of differential gene expression analysis on cholangiocytes between vehicle and seladelpar. Significantly upregulated and downregulated genes in the seladelpar group are shown in red dots. HSC, hepatic stellate cell; KC, Kupffer cell; LSEC, liver sinusoidal endothelial cell.

CD-1 mice were gavaged with vehicle or seladelpar (10 mg/kg body weight), and the liver was harvested 6 h later (Figure 1a). snRNA-seq analysis was performed, and clusters were labeled based on the markers derived from literature (Figure 1b). snRNA-seq shows the engagement of PPARD target genes primarily in hepatocytes and cholangiocytes by seladelpar (Figure 1c). The number of significantly differentially regulated genes by treatment with seladelpar was 733 (upregulated: 560; downregulated: 173) in hepatocytes, 66 in cholangiocytes (up: 51; down: 15), 2 in KCs (up: 2; down: 0), 3 in HSCs (up: 2; down: 1), and 0 in liver sinusoidal endothelial cells (LSECs). The top 20 up- or downregulated genes in each cell type are listed in Table S1. *Pdk4* and *Angptl4*, which are known PPARD target genes [8, 24], were significantly induced in hepatocytes. Among bile acid transporter genes, *Abcb4* was significantly upregulated in hepatocytes (Log_2_ fold change: 1.830; adjusted *p* value: 1.40e − 18). Figure 1c shows a heat map of the top 20 differentially regulated genes in each cell type. Most of these genes were differentially regulated in both hepatocytes and cholangiocytes. In particular, the top two genes induced in hepatocytes and cholangiocytes were *Ehhadh* and *Cyp4a14* (Figure 1d,e, Table S1). Ehhadh was also significantly induced in KCs. These genes are related to fatty acid metabolism [27, 28, 29]. On the other hand, cell-specific-induced genes were identified in KCs (Storkhead Box 2 (*Stox2*)) and HSCs (Erythroid Differentiation Regulator 1 y (*Gm47283*), MAM Domain Containing 2 (*Mamdc2*), and ABI Family Member 3 binding protein (Abi3bp)) (Figure S2). Taken together, seladelpar showed involvement in PPARD target genes mainly in hepatocytes and cholangiocytes, while limited specific gene induction was observed in KCs and HSCs.

We subsequently examined whether seladelpar caused different gene expression changes depending on hepatocyte zonation. Zone 1 and Zone 3 hepatocytes were defined using known marker genes (zone 1: Pck1, Hal, Gls2, and Arg1; zone 3: GS, Axin2, Cyp2e1, and OAT) (Figure S3a). A similar induction pattern was found in both zones, although some genes were specifically induced in Zone 1 or 3 (Figure S3b).

### 3.2. Bulk RNA Sequencing in Mouse Liver Confirms the Induction of Abcb4 in Hepatocytes by Seladelpar

**Figure 2:**
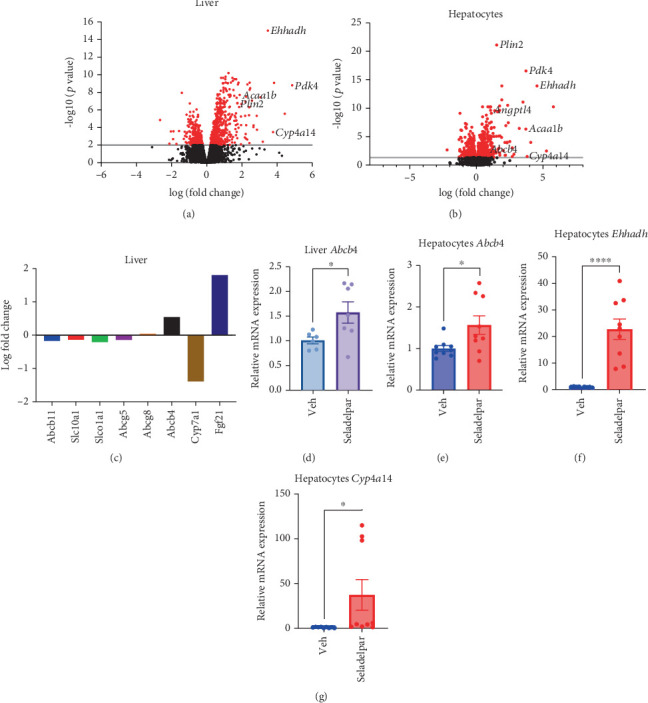
Bulk RNA sequencing in mouse liver. Male wild-type C57BL/6 mice were gavaged with vehicle (*n* = 6) or seladelpar (10 mg/kg body weight) (*n* = 7) and harvested after 6 h. (a) Volcano plot of differential gene expression analysis on mouse liver between vehicle and seladelpar. Significantly upregulated and downregulated genes in the seladelpar group are shown in red dots. (b) Volcano plot of differential gene expression analysis on mouse primary hepatocytes between vehicle and seladelpar. Significantly upregulated and downregulated genes in the seladelpar group are shown in red dots. (c) Alteration of bile acid metabolism–related gene expression in mouse liver following treatment with seladelpar. (d, e) *Abcb4* gene expression in mouse liver and primary hepatocytes by qPCR. Primary hepatocytes were treated with seladelpar (10 *μ*M) for 48 h. Results were obtained from two (c) or three (d) technical replicates. (f, g) *Ehhadh* and *Cyp4a14* gene expression in mouse liver and primary hepatocytes by qPCR. Primary hepatocytes were treated with seladelpar (10 *μ*M) for 48 h. Results were obtained from three technical replicates. Data are presented as mean ± S.E.M., ⁣^∗^*p* < 0.05, ⁣^∗∗^*p* < 0.01, and ⁣^∗∗^*p* < 0.0001 denote the significant difference between control and seladelpar.

We also performed gene expression analysis using liver samples from mice after oral administration of seladelpar (10 mg/kg body weight) after 6 h. The volcano plot of RNA sequencing from mouse liver samples confirmed the induction of genes, such as *Ehhadh* and *Cyp4a14,* as compared with the vehicle group, in addition to genes already known as PPARD target genes, such as *Pdk4* and Perilipin 2 (*Plin2*) (Figure 2a). We also performed bulk RNA sequencing using samples of isolated primary hepatocytes treated with seladelpar or vehicle for 48 h (Figure 2b). In addition to *Pdk4* and *Plin2*, *Ehhadh* and *Cyp4a14* were significantly induced, which is consistent with the snRNA sequencing results. The top 20 genes differentially regulated by seladelpar in primary hepatocytes are shown in Table S2. Given changes in genes involved in bile acid metabolism in our snRNA-seq analysis, we specifically analyzed changes in these genes. Among the bile acid transporter genes in mouse liver samples, *Abcb4* was significantly induced by seladelpar compared with the vehicle group (Figure 2c). As we have previously reported [8], *Fgf21* was induced, while Cytochrome P450, Family 7, Subfamily A, Member 1 (*Cyp7a1*) was suppressed.

We further confirmed the induction of *Abcb4* by seladelpar using qPCR in both mouse liver and primary hepatocytes (Figure 2d,e). *Ehhadh* and *Cyp4a14*, the top two genes in the snRNA-seq analysis, were induced by seladelpar in primary mouse hepatocytes using qPCR (Figure 2f,g).

### 3.3. Seladelpar Upregulates PPARD Target Gene Expression in Primary Mouse Cholangiocytes

**Figure 3:**
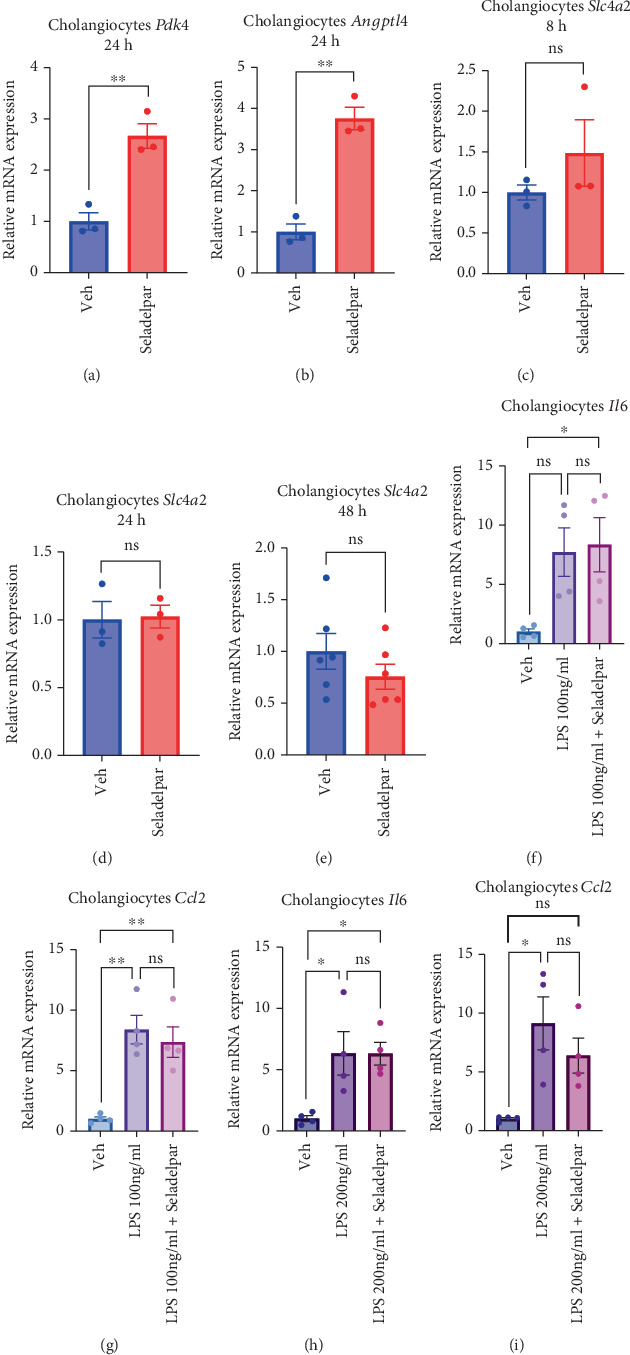
Effects of seladelpar on gene expression in primary mouse cholangiocytes. Primary cholangiocytes isolated from male wild-type C57BL/6 mice were treated with seladelpar (10 *μ*M) for 8, 24, and 48 h. LPS (100 or 200 ng/mL) was added in the presence or absence of seladelpar for 6 days. (a, b) *Pdk4* and *Angptl4* gene expression in mouse primary cholangiocytes. Results were obtained from three technical replicates. (c–e) *Slc4a2* gene expression in mouse cholangiocytes by seladelpar treatment time. Results were obtained from three technical replicates. (f, g) Gene expression of *Il6* and *Ccl2* by stimulation of mouse cholangiocytes with LPS (100 ng/mL, 6 days) and treatment with seladelpar (10 *μ*M, 6 days). Results were obtained from two technical replicates. (h, i) Gene expression of *Il6* and *Ccl2* by stimulation of mouse cholangiocytes with LPS (200 ng/mL, 6 days) and treatment with seladelpar (10 *μ*M, 6 days). Results were obtained from two technical replicates. Data are presented as mean ± S.E.M. ⁣^∗^*p* < 0.05 and ⁣^∗∗^*p* < 0.01 denote the significant difference between the groups. LPS, lipopolysaccharide.

Subsequently, we evaluated the effect of seladelpar on cultured intrahepatic cholangiocytes, which are important in the pathogenesis of chronic cholestatic liver diseases. PPARD target genes *Pdk4* and *Angptl4* were significantly upregulated in primary mouse cholangiocytes after 24 h of seladelpar treatment (Figure 3a,b). *Pdk4* and *Angptl4* were significantly upregulated at 48 h of treatment, but at 8 h, there was no significant difference, only an upward trend (Figure S4a–d). Under these conditions, the effect of seladelpar on *Slc4a2* (also known as AE2 or Anion Exchanger 2), an ion exchange molecule important in protection against bile duct injury [30], was evaluated. Slc4a2 facilitates a bicarbonate umbrella on the apical side of cholangiocytes, which protects them from bile acid–induced cell death [10]. *Slc4a2* expression was not significantly changed by seladelpar (Figures 3c, 3d, and 3e). We subsequently used LPS to induce inflammation in isolated primary cholangiocytes and evaluated the anti-inflammatory effects of seladelpar. *Il6* and *Ccl2*, cytokines and chemokines that play an important role in cholangiocyte inflammation, were significantly elevated in cultures with LPS [31] but were not significantly suppressed by seladelpar (Figures 3f, 3g, 3h, and 3i). *Ehhadh* and *Cyp4a14,* the top two genes in snRNA-seq, were not significantly induced by 24 h of seladelpar treatment in cultured primary mouse cholangiocytes (Figure S4e and S4f).

### 3.4. Seladelpar Upregulates PPARD Target Gene Expression in Primary Mouse KCs, but Does Not Significantly Suppress Inflammatory Gene Expression

**Figure 4:**
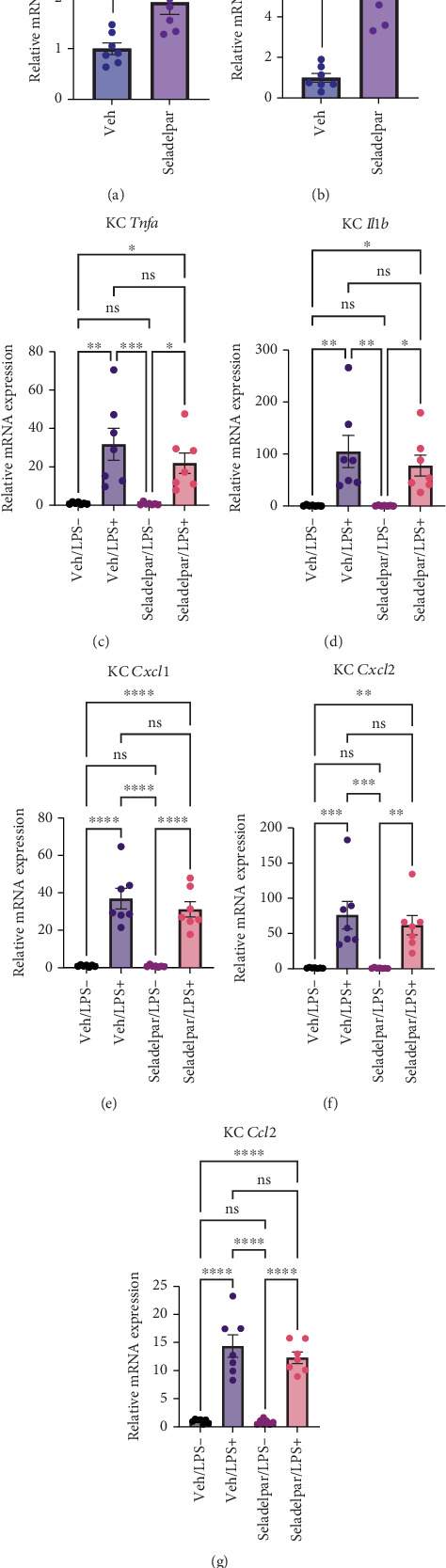
Effects of seladelpar on gene expression in primary mouse Kupffer cells. Primary KCs isolated from male wild-type C57BL/6 mice were incubated with seladelpar (10 *μ*M) for 2 h, before LPS (100 ng/mL) was added for an additional 6 h. (a, b) *Pdk4* and *Angptl4* gene expression in mouse primary KCs. Results were obtained from three technical replicates. (c–g) Gene expression of inflammatory genes by stimulation of mouse KCs with LPS (100 ng/mL, 6 h) and treatment with seladelpar (10 *μ*M). Results were obtained from three technical replicates. Data are presented as mean ± S.E.M. ⁣^∗^*p* < 0.05, ⁣^∗∗^*p* < 0.01, ⁣^∗∗∗^*p* < 0.001, and ⁣^∗∗∗∗^*p* < 0.0001 denote the significant difference between the groups. KC, Kupffer cell; LPS, lipopolysaccharide.

PPARD is also known to have anti-inflammatory effects on immune cells, especially macrophages [32, 33]. Therefore, we examined the effect of seladelpar on KCs, which are central to inflammation in the liver. We first confirmed the induction of PPARD target genes in primary mouse KCs following treatment with seladelpar for 8 h. *Pdk4* and *Angptl4* were significantly upregulated by treatment with seladelpar (Figure 4a,b). This suggests that seladelpar works well under our experimental conditions in primary KCs. Inflammatory-mediated gene expression, such as *Tnfa*, *Il1b*, *Cxcl1*, *Cxcl2*, and Ccl2, was subsequently measured to see if seladelpar could suppress LPS-induced inflammatory gene expression in KCs. As shown in Figures 4c, 4d, 4e, 4f, and 4g, no significant decrease in the expression of these genes by seladelpar was observed. *Ehhadh* was not significantly induced by seladelpar in cultured primary mouse KCs (Figure S5a).

### 3.5. Seladelpar Upregulates PPARD Target Gene Expression in Primary Mouse HSCs, but Does Not Suppress the Activation and Fibrogenic Potential

**Figure 5:**
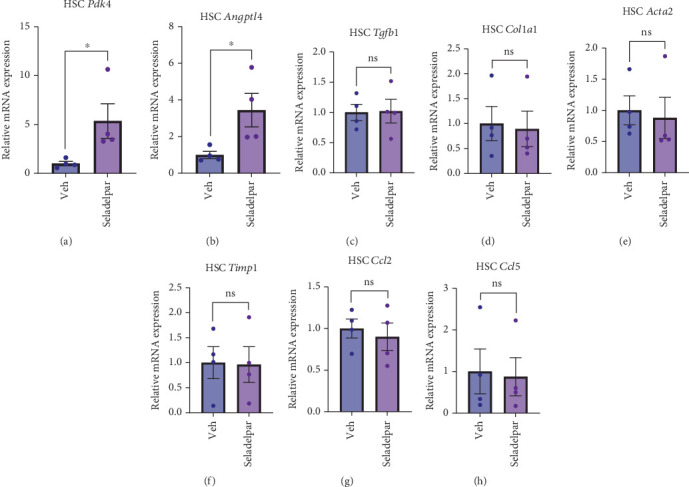
Effects of seladelpar on gene expression in primary mouse hepatic stellate cells. Primary HSCs isolated from male wild-type C57BL/6 mice were incubated with seladelpar (10 *μ*M) for 48 h. (a, b) *Pdk4* and *Angptl4* gene expression in primary mouse HSCs. Results were obtained from two technical replicates. (c–f) Fibrosis-related gene expression in mouse primary HSCs. Results were obtained from two technical replicates. (g, h) Inflammatory chemokine gene expression in mouse primary HSCs. Results were obtained from two technical replicates. Data are presented as mean ± S.E.M. ⁣^∗^*p* < 0.05 denotes the significant difference between the groups. HSC, hepatic stellate cell.

The effect of seladelpar on HSCs, the main fibrotic cell type during liver fibrosis, was also evaluated. When HSCs are isolated from the liver, they spontaneously activate on plastic in culture and upregulate fibrosis-related genes [34, 35]. We first confirmed the induction of the PPARD target genes in primary mouse HSCs after incubation with seladelpar for 48 h. *PDK4* and *Angptl4* were significantly upregulated by seladelpar in HSCs (Figure 5a,b). Under these conditions, we subsequently examined the effect of seladelpar on fibrosis-related gene expression, such as *Tgfb1*, *Col1a1*, *Acta2*, and *Timp1*, but found no significant effect of seladelpar on any of these genes (Figures 5c, 5d, 5e, and 5f). Inflammatory chemokines were also evaluated, but neither *Ccl2* nor *Ccl5* were significantly decreased by seladelpar (Figure 5g,h). We confirmed that seladelpar does not affect TGF-*β*1-induced gene expression of *COL1A1* and *ACTA2* in primary human HSCs (Figure S5b and S5c).

## 4. Discussion

In this study, we investigated the effects of the selective PPARD agonist seladelpar on liver cells using snRNA-seq analysis, bulk RNA sequencing, and primary cell culture. One of the important results of this study is that snRNA-seq shows the engagement of PPARD target genes primarily in hepatocytes and cholangiocytes by seladelpar. Our comprehensive analysis identified genes involved in fatty acid metabolism, such as *Ehhadh* and *Cyp4a14*, that were the top induced genes and upregulated both in hepatocytes and cholangiocytes, while some genes were induced in a cell type–specific manner. Also, significant induction of PPARD target genes represented by *Pdk4* and *Angptl4* was observed in all of the cells examined in this study, suggesting that all liver cells are potential therapeutic targets for seladelpar. The binding of PPARs to the promoter regions of *Pdk4* and *Angptl4* genes has been demonstrated in previous studies using ChIP-qPCR [36, 37], and our qPCR results reflect that in liver cells.

Interestingly, in the snRNA-seq analysis, PPARD target genes such as *Pdk4* and *Angptl4* [8, 24] were among the top 20 induced genes only in hepatocytes, but *Ehhadh* and *Cyp4a14* were strongly induced in three cell types (hepatocytes, cholangiocytes, and KCs). Cyp4a14 belongs to the Cytochrome P450 4 (CYP4) family and catalyzes omega-hydroxylation of saturated, branched-chain, and unsaturated fatty acids [27]. In relation to liver disease, *Cyp4a14* is elevated in the liver of a metabolic dysfunction–associated steatohepatitis (MASH) mouse model and is an alternative trigger of oxidative stress [38, 39, 40]. Furthermore, its deficiency significantly reduced liver injury, inflammation, and fibrosis in a methionine- and choline-deficient diet–induced MASH mouse model [41]. On the other hand, *Cyp4a14* gene overexpression reduced liver fibrosis markers such as *Tgfb1* and *Acta2* in a model of cholestatic liver fibrosis [42]. Ehhadh is involved in the peroxisomal fatty acid oxidation pathway [28, 29]. This gene is expressed in the liver and kidney, but its function in the liver and involvement in liver disease are still unknown [43]. Although the expression of *Ehhadh* and *Cyp4a14* is known to be induced by PPARA [29, 44], the strong induction by the selective PPARD agonist, seladelpar, is a new finding, and further studies are required to determine the effects of the drug on these genes and related pathways. Activation of PPARD has been shown to enhance fatty acid oxidation in hepatocytes, and this may, in turn, induce activation of PPARA, which also promotes fatty acid catabolism, resulting in a synergistic effect [45, 46]. Our present findings may therefore reflect an indirect activation of PPARA by seladelpar-induced PPARD activation. However, studies using knockout mice have demonstrated that PPARA and PPARD regulate distinct sets of genes under different physiological conditions [47]. It is also possible that pharmacological activation of PPARDs, as in this study, may result in transcriptional responses different from those observed with physiological activation. Interestingly, neither of these two genes was induced when isolated cholangiocytes or KCs were treated with seladelpar in culture. This might indicate that intercellular communication is important for seladelpar to exert at least some of its effects.

Known effects of PPARD agonism on hepatocytes include increasing fatty acid oxidation, regulating energy metabolism, and ameliorating insulin resistance [46, 48]. It is also involved in the production and secretion of bile acids in hepatocytes, and we have previously reported that seladelpar suppresses bile acid synthesis by decreasing hepatocyte CYP7A1 through the FGF21 signaling pathway [8]. This is consistent with our RNA sequencing results and the clinical effects of seladelpar in PBC. In addition, our data showed that the *Abcb4* gene, an important transporter for phospholipids from hepatocytes into canaliculi [49], was significantly upregulated by seladelpar. Increased micellar phospholipid concentrations render bile acids less toxic, which could contribute to the beneficial effect of seladelpar in PBC. PPARA is known to activate transcription of human *ABCB4* and to increase rat biliary phosphatidylcholine secretion [50]. PPARD may also be directly or indirectly involved in this pathway. ABCB4 is well known to show mutations in patients with progressive familial intrahepatic cholestasis (PFIC3), but there are also reports of association with cholestatic diseases such as primary sclerosing cholangitis (PSC) and PBC [51, 52, 53, 54].

Another important cell type in cholestatic liver disease is the cholangiocyte, but the direct effect of PPARD on cholangiocytes is not known. Xia et al. have reported that liver X receptor (LXR) beta and PPARD play a critical role in cholestatic liver diseases by regulating cholesterol flux from bile through cholangiocytes in a Niemann–Pick C1-Like L1 (NPC1L1)/ATP-binding Cassette Transporter A1 (ABCA1)–dependent manner [55]. In addition, from the perspective of lipid metabolism in cholangiocytes, the lipid mediator Sphingosine-1-Phosphate Receptor 2 (S1PR2) plays an important role in bile acid–induced cholangiocyte proliferation and liver injury in mice [56]. In our snRNA-seq analysis, genes related to lipid metabolism, such as *Ehhadh* and *Cyp4a14*, were strongly induced in cholangiocytes, suggesting that PPARD may exert functional effects on cholangiocytes via regulation of lipid metabolism. Further investigation of the function of these molecules in cholangiocytes and their relationship to PPARs is required. In addition, we did not find a significant inhibition of LPS-induced inflammatory gene regulation in KCs. Several anti-inflammatory effects via inhibition of NF*κ*B signaling by PPARA and PPARD agonists have been reported [57, 58]. The effect of PPARD agonists on liver fibrosis is a robust finding in mice [24]; however, the evidence for the reduction of liver fibrosis in humans remains unestablished [24, 59, 60].

This study has some limitations. Firstly, this study is based predominantly on mouse data. Secondly, LSECs have not been evaluated using primary cell culture. Third, we have not used mouse models of liver disease, such as MASH and PBC. Fourth, the consistency of induced genes in vivo and in vitro using RNA sequencing has only been studied in hepatocytes. Finally, although gender differences in PPAR expression have been reported [61], only male mice were used in this study.

In conclusion, seladelpar showed PPARD gene expression engagement across parenchymal and nonparenchymal liver cells. Seladelpar upregulated *Abcb4* in hepatocytes, which might contribute to its clinical benefit in PBC.

## Data Availability

The data that support the findings of this study are available from the corresponding author upon reasonable request. Bulk RNA-seq data was deposited to the Sequence Read Archive (SRA) with the BioProject ID PRJNA1276273. snRNA-seq data are accessible at 10.7910/DVN/AMAVSY.

## Supporting Information

Supporting Information 1 Table S1. Top 20 genes differentially regulated by seladelpar in each cell type (single-nuclei RNA sequencing). (a) Hepatocytes, (b) cholangiocytes, (c) Kupffer cells, and (d) hepatic stellate cells.

Supporting Information 2 Table S2. Top 20 genes differentially regulated by seladelpar in primary hepatocytes (bulk RNA sequencing).

Supporting Information 3 Figure S1. Evaluation of primary mouse cholangiocytes after isolation and magnetic cell sorting enrichment. (a) The Ep-CAM-positive cell rate of cholangiocyte samples after six passages was confirmed by fluorescence-activated cell sorting. Approximately 80% of the samples were Ep-CAM-positive. (b–d) Expression of cell-specific genes in hepatocytes (*Abcb4*) and cholangiocytes (*Krt19* and *Krt7*). Results were obtained from three technical replicates. Data are presented as mean ± S.E.M. ⁣^∗∗∗^*p* < 0.001 and ⁣^∗∗∗∗^*p* < 0.0001 denote the significant difference between the groups.

Supporting Information 4 Figure S2. Volcano plot of differential gene expression analysis on (a) KCs, (b) HSCs, and (c) LSECs between vehicle and seladelpar. Significantly upregulated and downregulated genes in the seladelpar group are shown in red dots. HSC, hepatic stellate cell; KC, Kupffer cell; LSEC, liver sinusoidal endothelial cell.

Supporting Information 5 Figure S3. Gene expression of Zone 1 and Zone 3 hepatocytes upon seladelpar treatment. (a) Zone 1 and Zone 3 hepatocytes were defined using known marker genes (Zone 1: Pck1, Hal, Gls2, and Arg1; Zone 3: GS, Axin2, Cyp2e1, and OAT). (b) Scatterplot of genes induced by seladelpar in Zone 1 to Zone 3. Three hundred and thirty-eight genes were significantly induced in both Zone 1 and Zone 3 (adj *p* < 0.05; purple dots), 257 genes were specifically induced in Zone 1 (blue dots), and 254 genes were specifically induced in Zone 3 (red dots).

Supporting Information 6 Figure S4. Gene expression in primary mouse cholangiocytes. Primary cholangiocytes isolated from male wild-type C57BL/6 mice were treated with seladelpar (10 *μ*M) for 8 and 48 h. (a–d) *Pdk4* and *Angptl4* gene expression in primary cholangiocytes at 8 and 48 h. Results were obtained from three technical replicates. (e, f) *Ehhadh* and *Cyp4a14* gene expression in primary cholangiocytes at 24 h. Results were obtained from three (e) or two (f) technical replicates. Data are presented as mean ± S.E.M. ⁣^∗∗^*p* < 0.01 denotes the significant difference between the groups.

Supporting Information 7 Figure S5. Gene expression in primary mouse Kupffer cells and primary human HSCs. (a) Primary KCs isolated from male wild-type C57BL/6 mice were incubated with seladelpar (10 *μ*M) for 8 h. *Ehhadh* gene expression in primary KCs. Results were obtained from three technical replicates. (b, c) Human HSCs were pretreated with 10 *μ*M seladelpar for 2 h prior to stimulation. TGF-*β*1 stimulation (5 ng/mL) was performed for 24 h. *COL1A1* and *ACTA2* gene expression in primary human HSCs was detected by qPCR. Data are presented as mean ± S.E.M. ⁣^∗∗∗^*p* < 0.001 and ⁣^∗∗∗∗^*p* < 0.0001 denote the significant difference between the groups. KC, Kupffer cell; HSC, hepatic stellate cell.
