# Supplementary material for: Single-Nuclei RNA Sequencing Shows the Engagement of PPAR-Delta Target Genes Primarily in Hepatocytes and Cholangiocytes by the Selective PPAR-Delta Agonist Seladelpar
Source: PPAR Res. 2025 Oct 23;2025:2935230. doi: 10.1155/ppar/2935230 (PMC12575037; doi:10.1155/ppar/2935230)
Supplement: Supporting Information 1 — Table S1. Top 20 genes differentially regulated by seladelpar in each cell type (single-nuclei RNA sequencing). (a) Hepatocytes, (b) cholangiocytes, (c) Kupffer cells, and (d) hepatic stellate cells. [file 2935230.f1.docx]

**Supplementary Table 1a. Top 20 genes differentially regulated by seladelpar in hepatocytes**

| Up or Down regulated | | |
| --- | --- | --- |
| Genes | Log_2_ fold change | adjusted p-value |
| Ehhadh | 5.84994 | 3.40E-82 |
| Cyp4a14 | 5.051097 | 3.45E-33 |
| Gm15441 | 4.736636 | 6.12E-100 |
| Cyp4a10 | 4.403894 | 5.47E-12 |
| Acaa1b | 3.983249 | 3.99E-73 |
| Aldh3a2 | 3.672238 | 1.65E-162 |
| Acot1 | 3.44647 | 6.69E-115 |
| Pex11a | 3.273464 | 5.01E-235 |
| Retsat | 3.221989 | 2.92E-11 |
| Pdk4 | 2.933929 | 2.87E-32 |
| Plin2 | 2.879247 | 1.02E-84 |
| Acox1 | 2.761034 | 2.79E-63 |
| Hsd17b11 | 2.729747 | 4.92E-221 |
| Cbfa2t3 | 2.667477 | 1.03E-08 |
| Hmgcs2 | 2.570693 | 5.05E-45 |
| Angptl4 | 2.499432 | 2.06E-77 |
| Fabp1 | 2.48889 | 8.26E-36 |
| Cyp2c70 | -2.47241 | 1.85E-05 |
| Ephx1 | 2.47121 | 8.09E-45 |
| Slc22a5 | 2.445471 | 2.15E-113 |

**Supplementary Table 1b. Top 20 genes differentially regulated by seladelpar in cholangiocytes**

| Up or Down regulated | | |
| --- | --- | --- |
| Genes | Log_2_ fold change | adjusted p-value |
| Ehhadh | 5.726092 | 3.26E-16 |
| Cyp4a14 | 4.940114 | 0.000491131 |
| Gm15441 | 4.40159 | 7.89E-14 |
| Cyp4a10 | 3.957651 | 3.75E-12 |
| Acaa1b | 3.404813 | 1.18E-33 |
| Aldh3a2 | 3.216213 | 0.019729165 |
| Acot1 | 3.163941 | 4.50E-07 |
| Hmgcs2 | 2.674871 | 1.17E-29 |
| Acox1 | 2.674228 | 1.30E-15 |
| Cyp2c70 | -2.66983 | 0.007067866 |
| Gfra1 | -2.6564 | 5.69E-09 |
| Plin2 | 2.458969 | 2.95E-10 |
| Pex11a | 2.351083 | 9.96E-11 |
| Fabp1 | 2.350937 | 1.17E-07 |
| B3galt1 | -2.3077 | 4.68E-06 |
| Hsd17b11 | 2.144437 | 6.07E-07 |
| Ephx2 | 2.099451 | 2.95E-17 |
| Lamb3 | 2.080309 | 0.001629138 |
| Acsl1 | 2.068799 | 1.31E-08 |
| Gm36264 | -2.043 | 0.000180765 |

**Supplementary Table 1c. Top 20 genes differentially regulated by seladelpar in Kupffer cells**

| Up regulated | | |
| --- | --- | --- |
| Genes | Log_2_ fold change | adjusted p-value |
| Ehhadh | 2.758028 | 0.019099 |
| Stox2 | 0.963325 | 0.005872 |

**Supplementary Table 1d. Top 20 genes differentially regulated by seladelpar in hepatic stellate cells**

| Up or Down regulated | | |
| --- | --- | --- |
| Genes | Log_2_ fold change | adjusted p-value |
| Gm47283 | -1.4688 | 6.27E-05 |
| Mamdc2 | 1.11853 | 0.017919 |
| Abi3bp | 0.869742 | 0.016303 |
