# Supplementary material for: Single-Nuclei RNA Sequencing Shows the Engagement of PPAR-Delta Target Genes Primarily in Hepatocytes and Cholangiocytes by the Selective PPAR-Delta Agonist Seladelpar
Source: PPAR Res. 2025 Oct 23;2025:2935230. doi: 10.1155/ppar/2935230 (PMC12575037; doi:10.1155/ppar/2935230)
Supplement: Supporting Information 2 — Table S2. Top 20 genes differentially regulated by seladelpar in primary hepatocytes (bulk RNA sequencing). [file 2935230.f2.docx]

**Supplementary Table 2. Top 20 genes differentially regulated by seladelpar in primary hepatocytes (Bulk RNA sequencing)**

| Up or Down regulated | | |
| --- | --- | --- |
| Genes | Log_2_ fold change | adjusted p-value |
| Acot1 | 5.784534 | 5.84E-11 |
| Acot3 | 5.262266 | 0.003484 |
| Ehhadh | 4.554845 | 1.22E-14 |
| Fbp2 | 4.085701 | 0.000104 |
| Cyp4a14 | 3.814834 | 0.031862 |
| Pdk4 | 3.727972 | 2.84E-17 |
| Acaa1b | 3.718748 | 4.88E-07 |
| Acot2 | 3.503166 | 8.59E-12 |
| Cpt1b | 3.222798 | 3.59E-07 |
| Cyp4a10 | 2.824716 | 0.012643 |
| Cyp4a31 | 2.700866 | 0.032067 |
| Ivl | 2.69788 | 0.000943 |
| Mgll | 2.516825 | 7.60E-05 |
| Plat | 2.398776 | 3.64E-08 |
| Ucp2 | 2.355248 | 3.14E-11 |
| Hmgcs2 | 2.313427 | 0.0001 |
| Gm4952 | 2.30247 | 0.000126 |
| Rab15 | 2.217593 | 1.19E-07 |
| Plcl1 | -2.19403 | 0.002221 |
| Fabp1 | 2.192479 | 0.018471 |
