# Supplementary figures and images for: Single-Nuclei RNA Sequencing Shows the Engagement of PPAR-Delta Target Genes Primarily in Hepatocytes and Cholangiocytes by the Selective PPAR-Delta Agonist Seladelpar
Source: PPAR Res. 2025 Oct 23;2025:2935230. doi: 10.1155/ppar/2935230 (PMC12575037; doi:10.1155/ppar/2935230)

Supplementary Figure 1

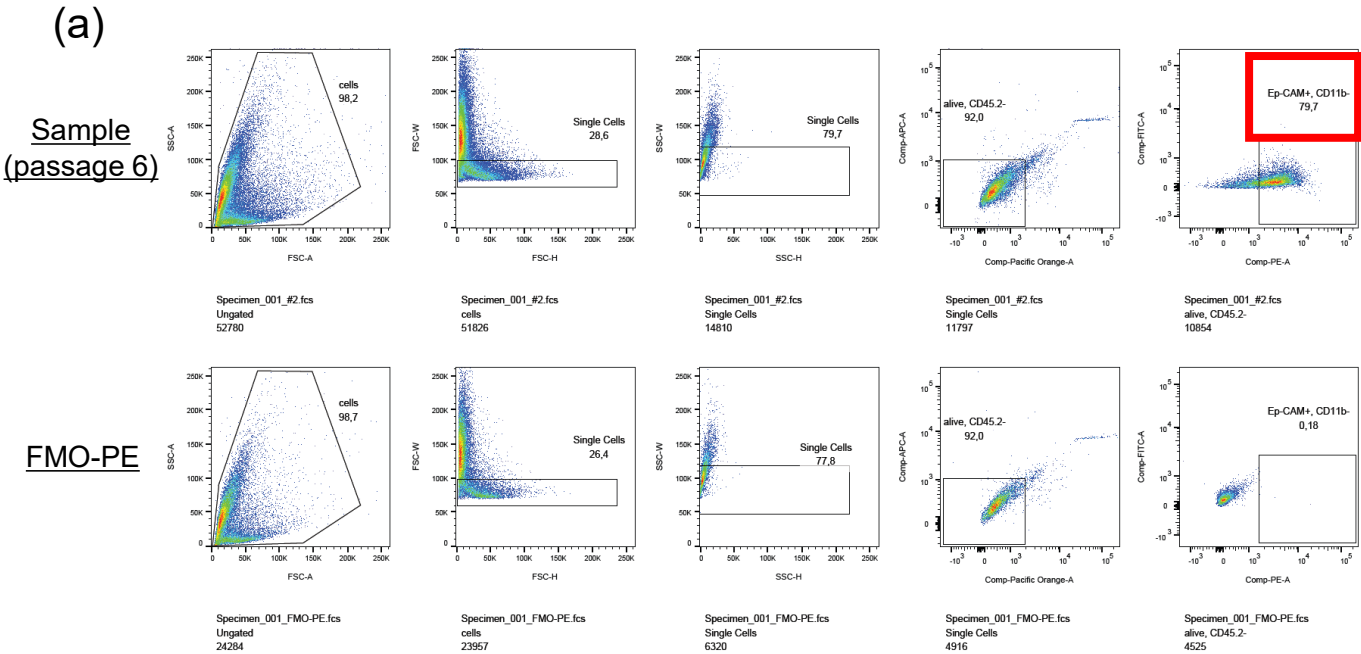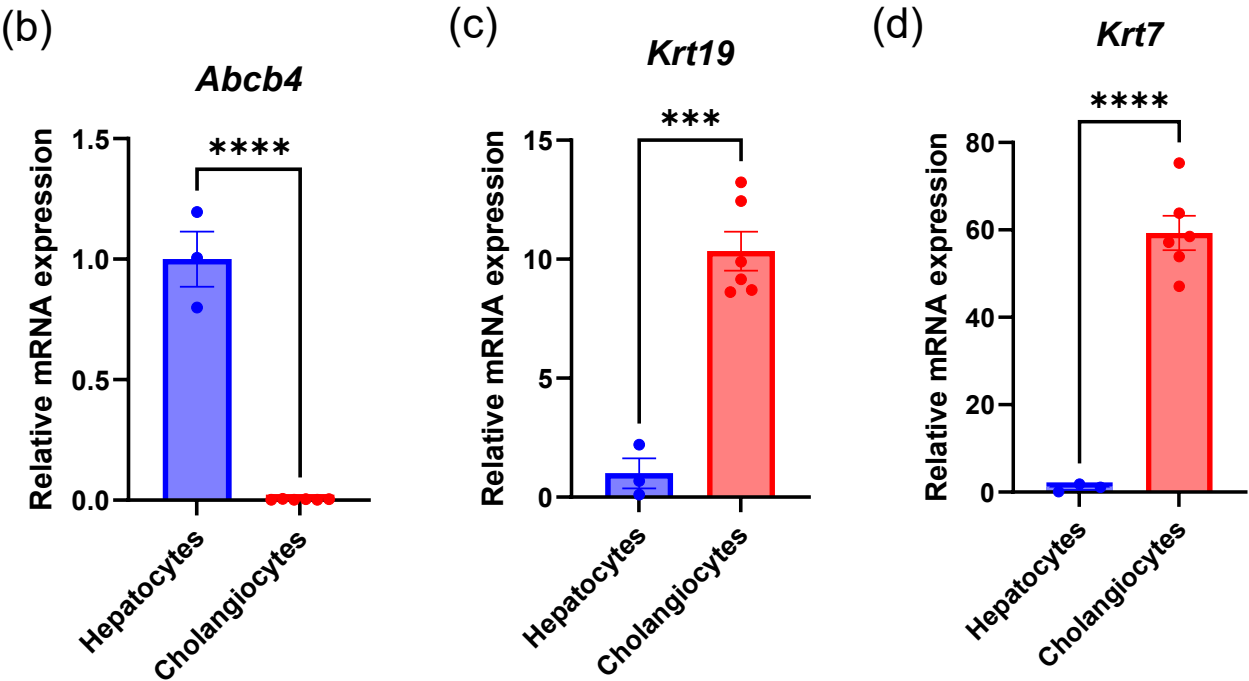

Supplement: Supporting Information 3 — Figure S1. Evaluation of primary mouse cholangiocytes after isolation and magnetic cell sorting enrichment. (a) The Ep-CAM-positive cell rate of cholangiocyte samples after six passages was confirmed by fluorescence-activated cell sorting. Approximately 80% of the samples were Ep-CAM-positive. (b–d) Expression of cell-specific genes in hepatocytes (Abcb4) and cholangiocytes (Krt19 and Krt7). Results were obtained from three technical replicates. Data are presented as mean ± S.E.M. ⁣∗∗∗p < 0.001 and ⁣∗∗∗∗p < 0.0001 denote the significant difference between the groups. [file 2935230.f3.pdf]

## Supplementary Figure 2

(a)

**KC**

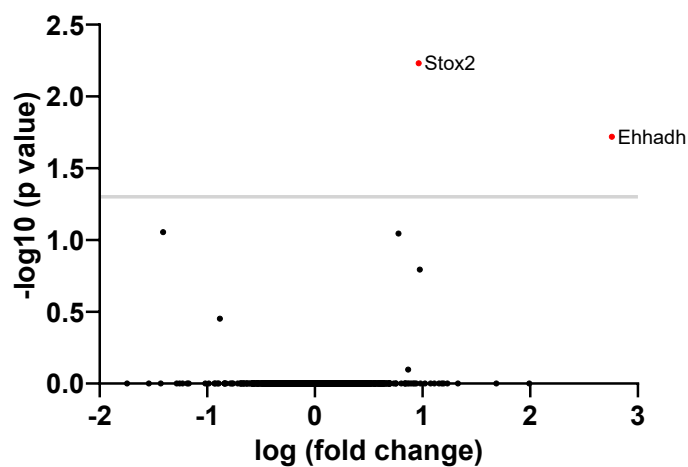

(b)

**HSC**

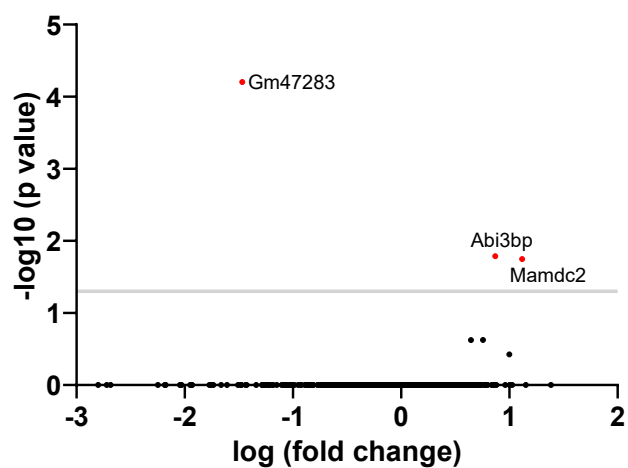

(c)

**LSEC**

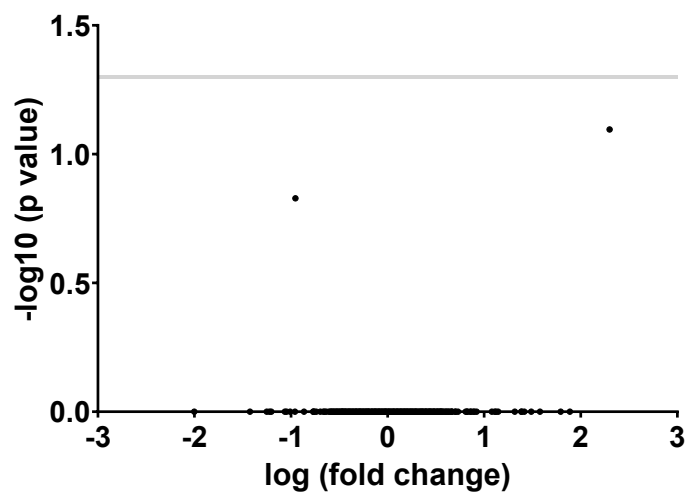

Supplement: Supporting Information 4 — Figure S2. Volcano plot of differential gene expression analysis on (a) KCs, (b) HSCs, and (c) LSECs between vehicle and seladelpar. Significantly upregulated and downregulated genes in the seladelpar group are shown in red dots. HSC, hepatic stellate cell; KC, Kupffer cell; LSEC, liver sinusoidal endothelial cell. [file 2935230.f4.pdf]

Supplementary Figure 3

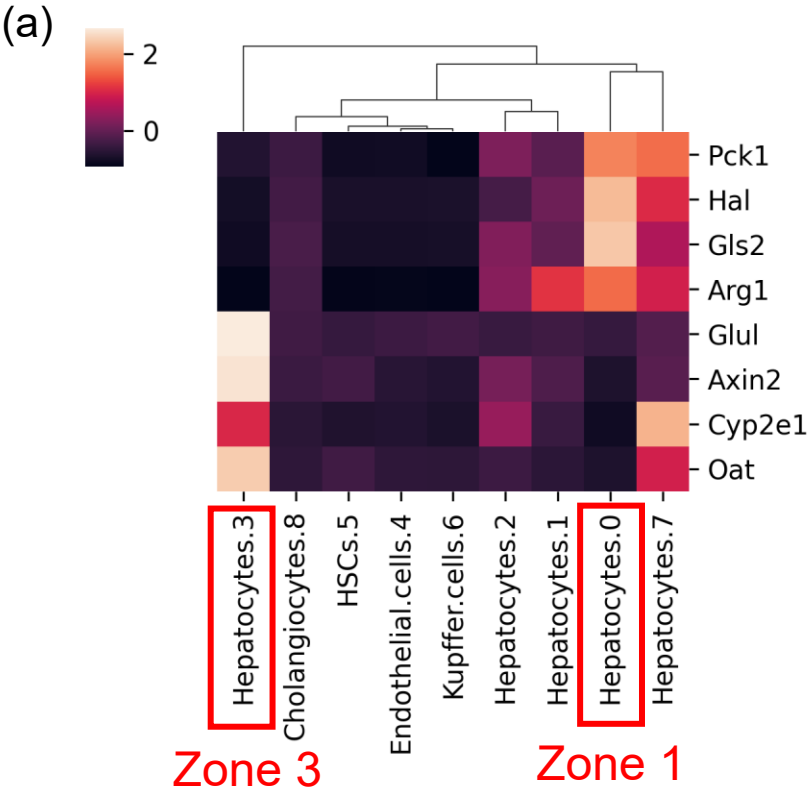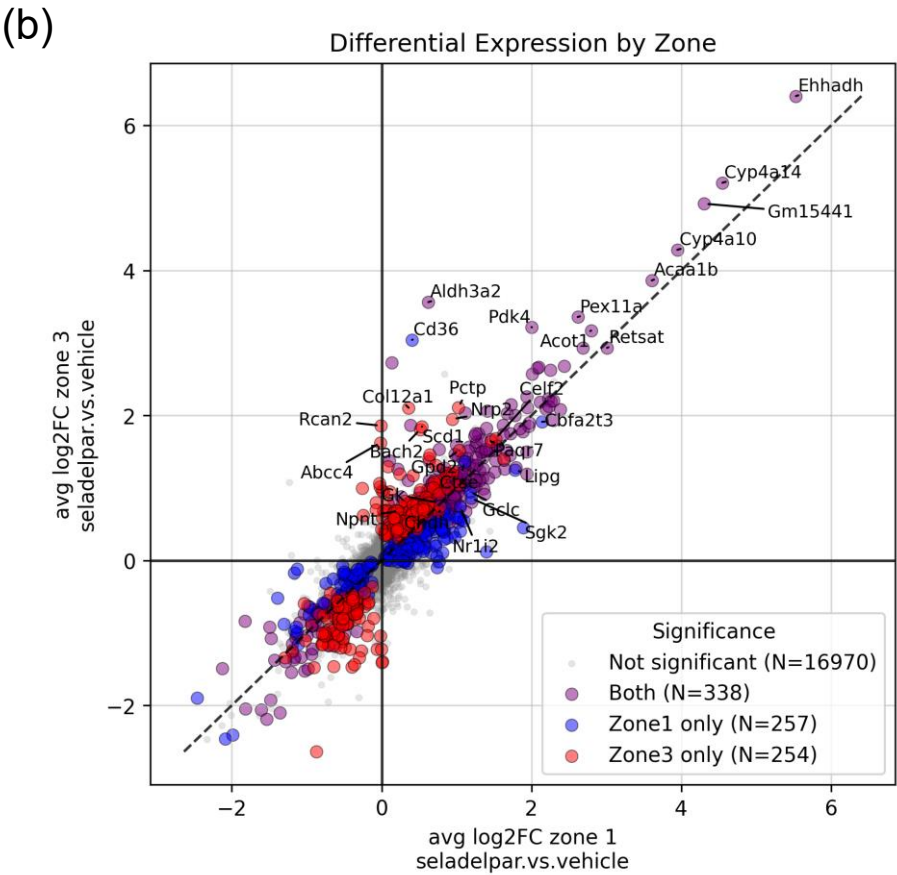

Supplement: Supporting Information 5 — Figure S3. Gene expression of Zone 1 and Zone 3 hepatocytes upon seladelpar treatment. (a) Zone 1 and Zone 3 hepatocytes were defined using known marker genes (Zone 1: Pck1, Hal, Gls2, and Arg1; Zone 3: GS, Axin2, Cyp2e1, and OAT). (b) Scatterplot of genes induced by seladelpar in Zone 1 to Zone 3. Three hundred and thirty-eight genes were significantly induced in both Zone 1 and Zone 3 (adj p < 0.05; purple dots), 257 genes were specifically induced in Zone 1 (blue dots), and 254 genes were specifically induced in Zone 3 (red dots). [file 2935230.f5.pdf]

Supplementary Figure 4

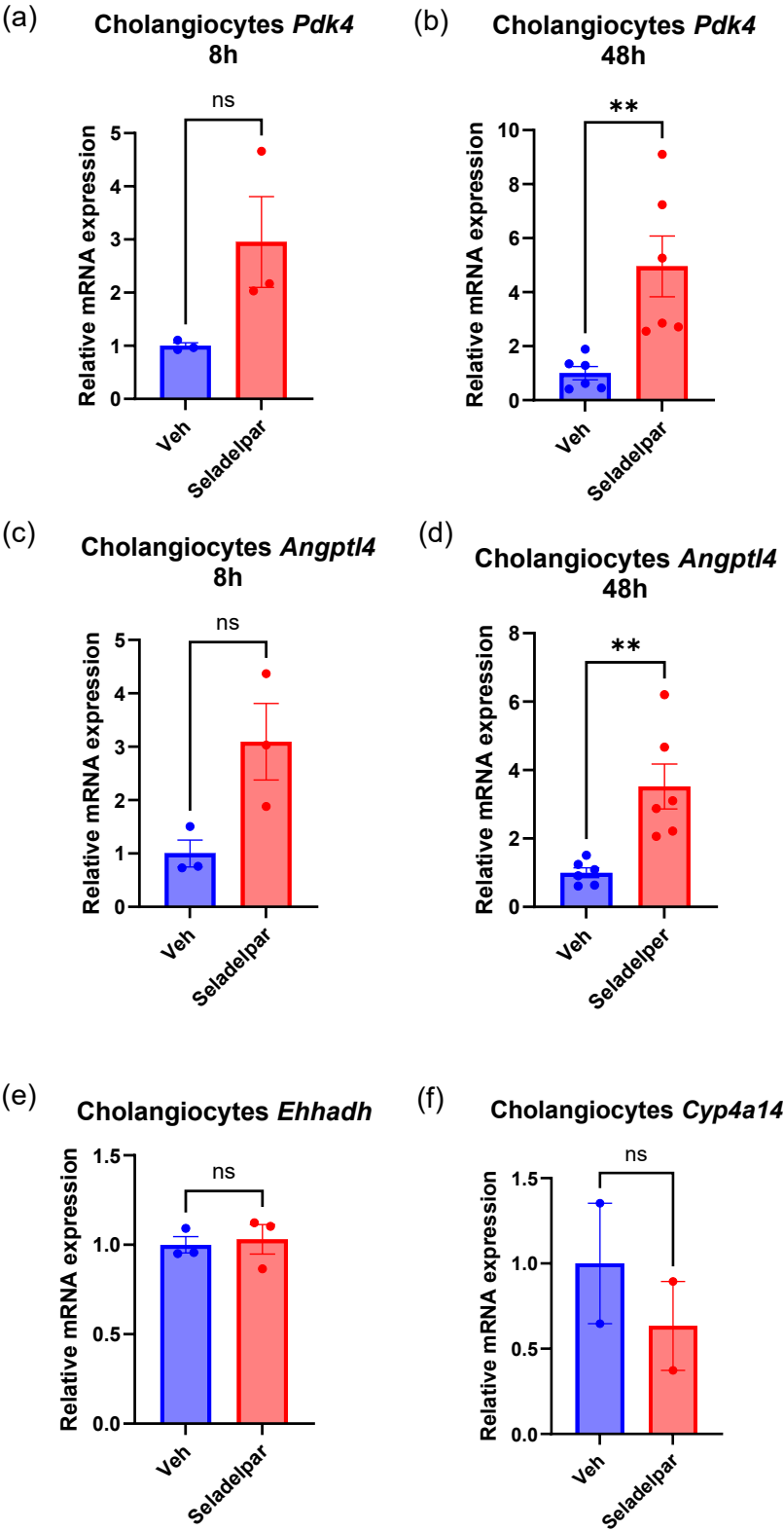

Supplement: Supporting Information 6 — Figure S4. Gene expression in primary mouse cholangiocytes. Primary cholangiocytes isolated from male wild-type C57BL/6 mice were treated with seladelpar (10 μM) for 8 and 48 h. (a–d) Pdk4 and Angptl4 gene expression in primary cholangiocytes at 8 and 48 h. Results were obtained from three technical replicates. (e, f) Ehhadh and Cyp4a14 gene expression in primary cholangiocytes at 24 h. Results were obtained from three (e) or two (f) technical replicates. Data are presented as mean ± S.E.M. ⁣∗∗p < 0.01 denotes the significant difference between the groups. [file 2935230.f6.pdf]

Supplementary Figure 5

(a)

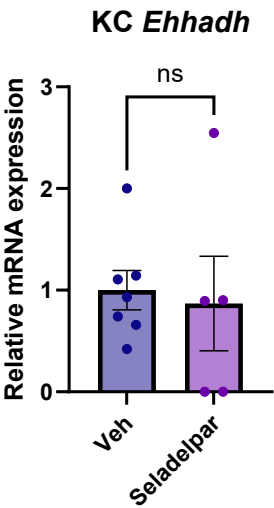

(b)

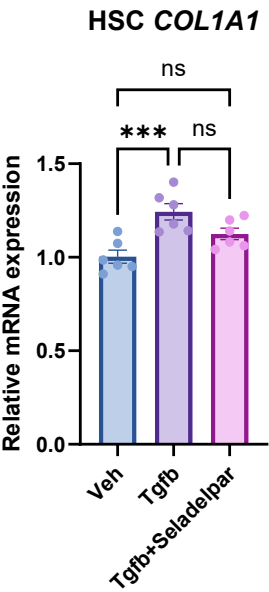

(c)

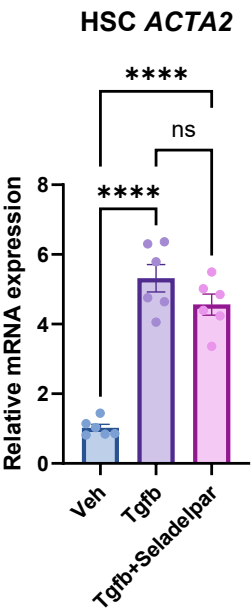

Supplement: Supporting Information 7 — Figure S5. Gene expression in primary mouse Kupffer cells and primary human HSCs. (a) Primary KCs isolated from male wild-type C57BL/6 mice were incubated with seladelpar (10 μM) for 8 h. Ehhadh gene expression in primary KCs. Results were obtained from three technical replicates. (b, c) Human HSCs were pretreated with 10 μM seladelpar for 2 h prior to stimulation. TGF-β1 stimulation (5 ng/mL) was performed for 24 h. COL1A1 and ACTA2 gene expression in primary human HSCs was detected by qPCR. Data are presented as mean ± S.E.M. ⁣∗∗∗p < 0.001 and ⁣∗∗∗∗p < 0.0001 denote the significant difference between the groups. KC, Kupffer cell; HSC, hepatic stellate cell. [file 2935230.f7.pdf]
